# The Accuracy of Digital vs Conventional Impression among Patients Receiving Dental Implants: A Systematic Review and Meta Analysis of Clinical Studies: Short title: Digital vs Conventional Impression among Patients Receiving Dental Implants

**DOI:** 10.31661/gmj.v15i.4212

**Published:** 2026-09-26

**Authors:** Sana Taherzadeh, Saman Kalantari

**Affiliations:** ^1^ AJA University of Medical Sciences, Tehran, Iran; ^2^ Tehran University of Medical Sciences, Tehran, Iran; ^3^ Department of Prosthodontics, School of Dentistry, Islamic Azad University of Medical Sciences, Tehran, Iran

**Keywords:** Digital Impression, Conventional Impression, Intraoral Scanner, Dental Implants, Distance Deviation, Angular Deviation

## Abstract

**Background:**

The accuracy of implant impressions is critical for achieving passive fitness and preventing biological and mechanical complications. Digital intraoral scanners have emerged as an alternative to conventional elastomeric impressions, but their comparative three-dimensional accuracy in partially and completely edentulous patients remains unclear.

**Materials and Methods:**

An electronic search of PubMed, Scopus, and Web of Science was performed. Clinical human studies directly comparing digital and conventional implant impressions in partially or completely edentulous patients were included. Distance- and angular-deviation were pooled.

**Results:**

Eight clinical studies were included. In the prespecified random-effects analysis, no significant difference was found between digital and conventional impressions for distance deviation (MD = 1.14 mm; 95% CI, -1.88 to 4.16; I2 = 98.7%) or angular deviation (MD = 0.12 degrees; 95% CI, -3.43 to 3.67; I2 = 97.5%). Fixed-effect estimates were calculated only as exploratory sensitivity analyses because heterogeneity was extreme. Subgroup findings, including lower distance and angular deviation with digital impressions in single-implant cases, should be interpreted cautiously because they were based on few studies and, for angular deviation, a single study.

**Conclusions:**

Digital and conventional impressions showed comparable overall three-dimensional accuracy under random-effects models; however, the certainty and generalizability of the pooled estimates are limited by extreme heterogeneity, sparse clinical evidence, and subgroup analyses with limited data. Digital workflows may be clinically useful for single-implant and short-span cases, whereas multi-implant and full-arch cases require individualized technique selection according to arch length, implant distribution, operator experience, and available verification methods.

## Introduction

Implant-supported prostheses have become a predictable option for restoring function and aesthetics in partially and completely edentulous patients, but their long-term success depends heavily on the accuracy of the implant impression. Any misfit at the implant–abutment or framework–implant interface can translate into biological and mechanical complications, including screw loosening, component fracture, and bone loss [1, 2]. Conventional impression techniques, typically based on elastomeric materials and open- or closed-tray procedures, have long been considered the reference standard. However, they are technique-sensitive and subject to dimensional changes related to tray rigidity, material shrinkage, and stone expansion, all of which may compromise the passive fit of the final restoration [3, 4]. Digital intraoral impression systems have been introduced as an alternative that may overcome some of these limitations by directly capturing the three-dimensional position of scan bodies and eliminating several laboratory steps. Beyond the promise of improved patient comfort and streamlined workflows, digital impressions might reduce distortion associated with impression materials and casts, particularly in single-unit and short-span implant restorations [5, 6]. Nevertheless, there is still debate regarding their accuracy in more complex clinical situations, such as multiple implants, long-span prostheses, and full-arch reconstructions. Experimental and clinical studies have reported conflicting findings, and the influence of factors such as edentulous status, the number of implants, and the extent of tooth loss on the relative performance of digital versus conventional impressions remains unclear [7, 8, 9]. Thus, the present study aimed to compare the three-dimensional accuracy of digital and conventional implant impressions in patients with partial or complete edentulism, using distance deviation and angular deviation as primary outcomes.

### Search Strategies and Data Synthesis

The review protocol was not prospectively registered in PROSPERO or another systematic-review registry. The search was limited to peer-reviewed articles indexed in PubMed, Scopus, and Web of Science. Grey literature, dissertations, and conference abstracts were not systematically searched. Study screening and eligibility assessment were performed independently by two reviewers, and disagreements were resolved through discussion with a third reviewer.

## Systematic Search

A comprehensive search was conducted in electronic databases, including Web of Science, Scopus, and PubMed, encompassing all relevant clinical studies up to January 2025. The search employed Medical Subject Headings (MeSH) and free-text keywords tailored to implant impressions and edentulism, using combinations of terms such as: (“dental implant*” OR “implant-supported” OR “implant”) AND (“digital impression” OR “intraoral scan*”) AND (“conventional impression” OR “analog impression” OR “elastomeric impression”) AND (“accuracy” OR “trueness” OR “precision” OR “distance deviation” OR “angular deviation”).

### Inclusion and Eligibility Criteria

Study eligibility was structured using the PICO framework as follows: Population (P): Partially or completely edentulous patients receiving dental implants, regardless of arch (maxilla or mandible), number of implants, or prosthesis type. Intervention (I): Digital impression techniques using intraoral scanners for the transfer of implant position. Comparison (C): Conventional (analog) implant impression techniques employing elastomeric materials (e.g., open-tray or closed-tray methods). Outcome (O): Three-dimensional accuracy outcomes expressed as distance deviation and angular deviation between the planned or reference implant position and the position captured by the impression technique. We included clinical human studies directly comparing digital and conventional impressions and reporting quantitative data for at least one of the predefined outcomes. Studies were excluded if they were in vitro or purely laboratory-based, case reports, animal studies, reviews, or if they lacked clear description of the impression technique, did not involve implant-supported restorations, or did not provide extractable data on distance or angular deviation.

### Risk of Bias Assessment

wo reviewers independently assessed the methodological quality and risk of bias of the included studies. Randomized clinical trials were evaluated using the revised Cochrane risk-of-bias tool for randomized trials (RoB 2), and nonrandomized or observational clinical studies were evaluated using ROBINS-I. Disagreements were resolved by consensus or consultation with a third reviewer. Judgments were summarized at the study level and across domains, and the results were presented in a risk-of-bias table and figure. The influence of study quality was considered when interpreting pooled estimates, subgroup findings, and the overall certainty of conclusions.

### Data Extraction and Outcome Measures

Two independent reviewers extracted relevant data using a standardized extraction form, and any disagreements were resolved through discussion with a third reviewer. Extracted variables included first author’s name, year of publication, study design, patient demographics, sample size, type of edentulism (partial or complete), number and distribution of implants, number of missing teeth, arch involved (maxilla and/or mandible), details of the digital and conventional impression techniques, reference method used to assess accuracy, and quantitative outcomes for distance and angular deviation (means, standard deviations, and sample sizes for each group).

### Statistical Analysis and Data Synthesis

The pooled effects for distance deviation and angular deviation between digital and conventional impressions were calculated as mean differences (MDs; digital minus conventional) with corresponding 95% confidence intervals. Given the expected clinical and methodological variability among studies, random-effects models were used as the primary approach to account for between-study heterogeneity, and fixed-effect models were calculated in parallel for comparison. Statistical heterogeneity was quantified using the I² statistic, with values greater than 50% considered indicative of substantial heterogeneity. Forest plots were generated to visually summarize individual study effects and pooled estimates for each outcome. Funnel plots were used to visually explore potential publication bias. Predefined subgroup analyses were performed according to the number of implants (single vs multiple), edentulous status (partial vs complete), and extent of tooth loss (<10 vs >10 missing teeth). Sensitivity analyses were conducted by sequentially omitting individual studies and by restricting the analyses to specific subgroups to assess the robustness of the pooled results. All statistical analyses were performed using the meta package in R (R Foundation for Statistical Computing, Vienna, Austria) within the RStudio environment (RStudio Inc., Boston, MA). Quantitative pooling was retained because the included studies addressed the same PICO question, directly compared digital and conventional impression techniques, and reported comparable continuous accuracy outcomes. Nevertheless, important clinical and methodological diversity was expected because studies differed in implant number, edentulous status, arch, scanner system, scan body, conventional impression technique, reference standard, and outcome-measurement protocol. Therefore, random-effects models were prespecified as the primary analysis. Fixed-effect models were calculated only as exploratory sensitivity analyses and were not used as the basis for clinical conclusions when heterogeneity was substantial or extreme. Meta-regression was considered but was not performed because the number of included comparisons was too small to support reliable covariate modelling. Publication bias was explored visually using funnel plots when feasible. Formal small-study-effect testing, such as Egger’s test, was not performed because fewer than 10 comparisons were available for each primary outcome, making such tests underpowered and potentially misleading.

## Results

**Table:** Table1. Detailed Characteristics of the included studies

									Distance Deviation				Angular Deviation		
Author	Year	Country	Design	N	Type	CE	TL	Conventional	Digital	Conventional	Digital				
								M	SD	M	SD	M	SD	M	SD
Andriessen [11]	2014	Netherlands	CSS	25	Multiple	Yes	>10	20.2	4.02	20.21	4.09	8.61	3.94	8.4	4.45
Alsharbaty [10]	2018	Iran	CSS	36	Multiple	No	<10	90	20	220	30	2.01	0.33	6.77	0.91
Jiang [15]	2019	China	PCS	31	Multiple	No	<10	14.65	4.64	27.43	13.47	-	-	-	-
Tallarico [17]	2019	Italy	RCT	90	Multiple	No	<10	0.52	0.3	0.61	0.49	2.25	1.41	1.98	2.38
Nagata [16]	2021	Japan	PCS	30	Single	No	>10	50.7	14.9	45.4	13.4	-	-	-	-
Dohiem [12]	2022	Egypt	RCT	8	Multiple	No	<10	47.79	4.6	21.45	3.3	-	-	-	-
								40.04	4.1	21.45	3.3	-	-	-	-
Hanozin [14]	2022	Belgium	RCT	18	Single	No	<10	1.27	0.83	0.65	0.37	6.26	3.97	2.41	1.2
Elashry [13]	2024	Egypt	RCT	8	Multiple	No	>10	15.2	2.13	15.18	0.96	2.34	1.71	2.24	1.06
								17.02	3.73	16.91	3.74	-	-	-	-

**TL:** Number of lost teeth;**CE:** Complete edentulism;**SD:** Standard deviation; **M:** Mean

Our initial search yielded 1,096 articles from PubMed, Scopus, and Web of Science, from which we eliminated 490 duplicates. After reviewing the titles and abstracts of the remaining 606 records, we retrieved 52 full-text articles for further evaluation. Ultimately, 8 studies [10, 11, 12, 13, 14, 15, 16, 17] met our eligibility criteria and were included in the systematic review and meta-analysis (Figure-1). Detailed characteristics of the included studies are summarized in Table1.

**Figure-1:**
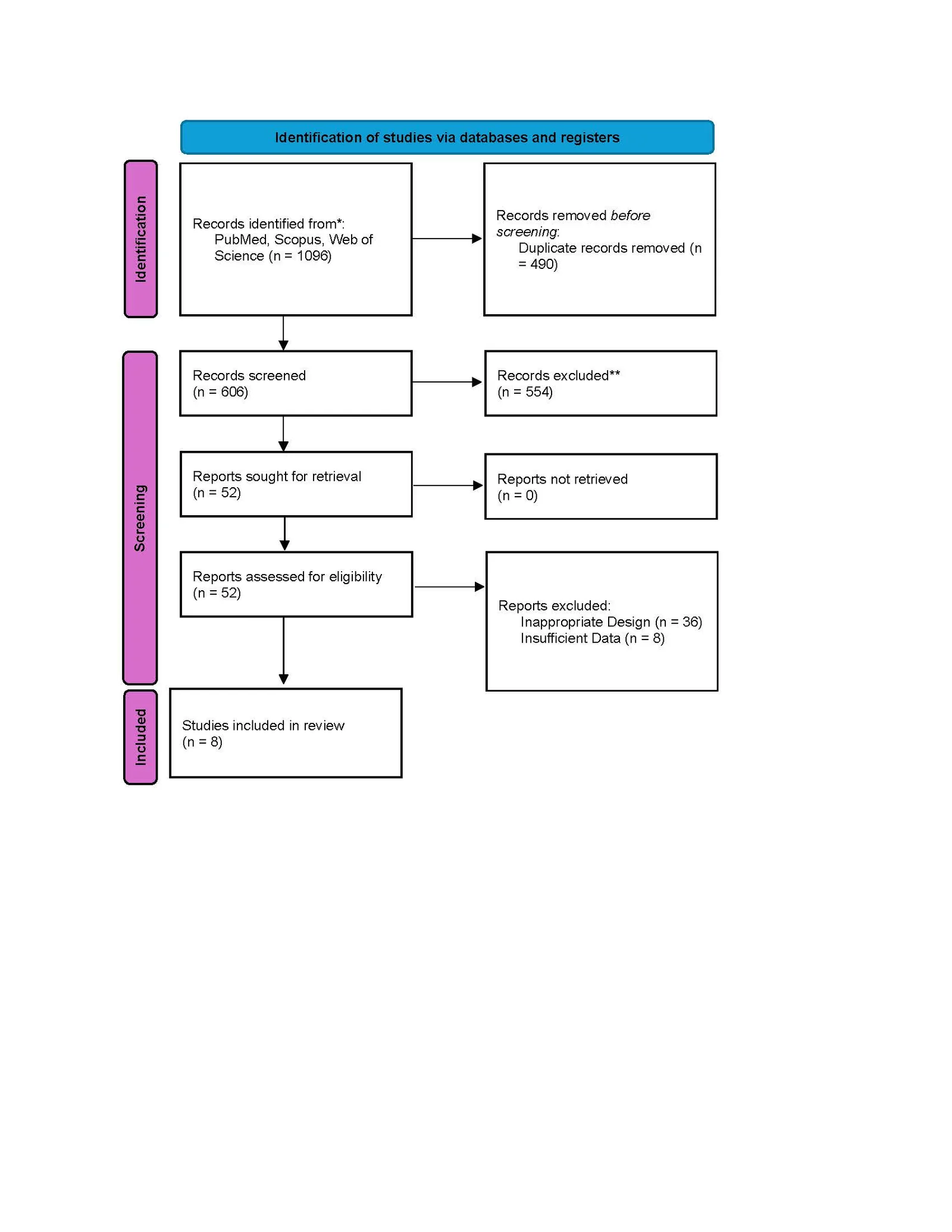
Figure 1. PRISMA flow diagram of the included studies

**Figure-2:**
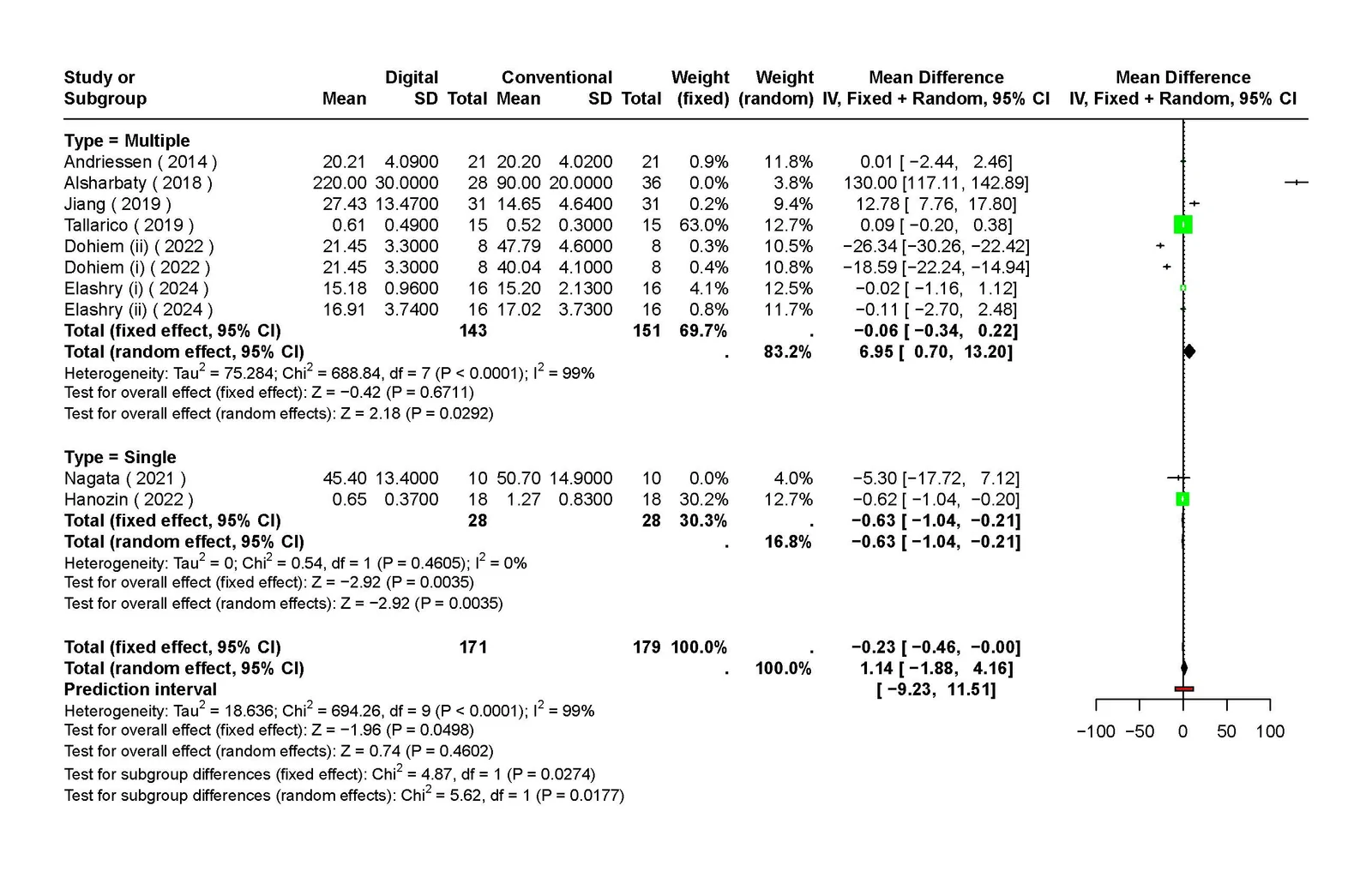
Figure 2. Forest plot of distance deviation based on number of implants

**Figure-3:**
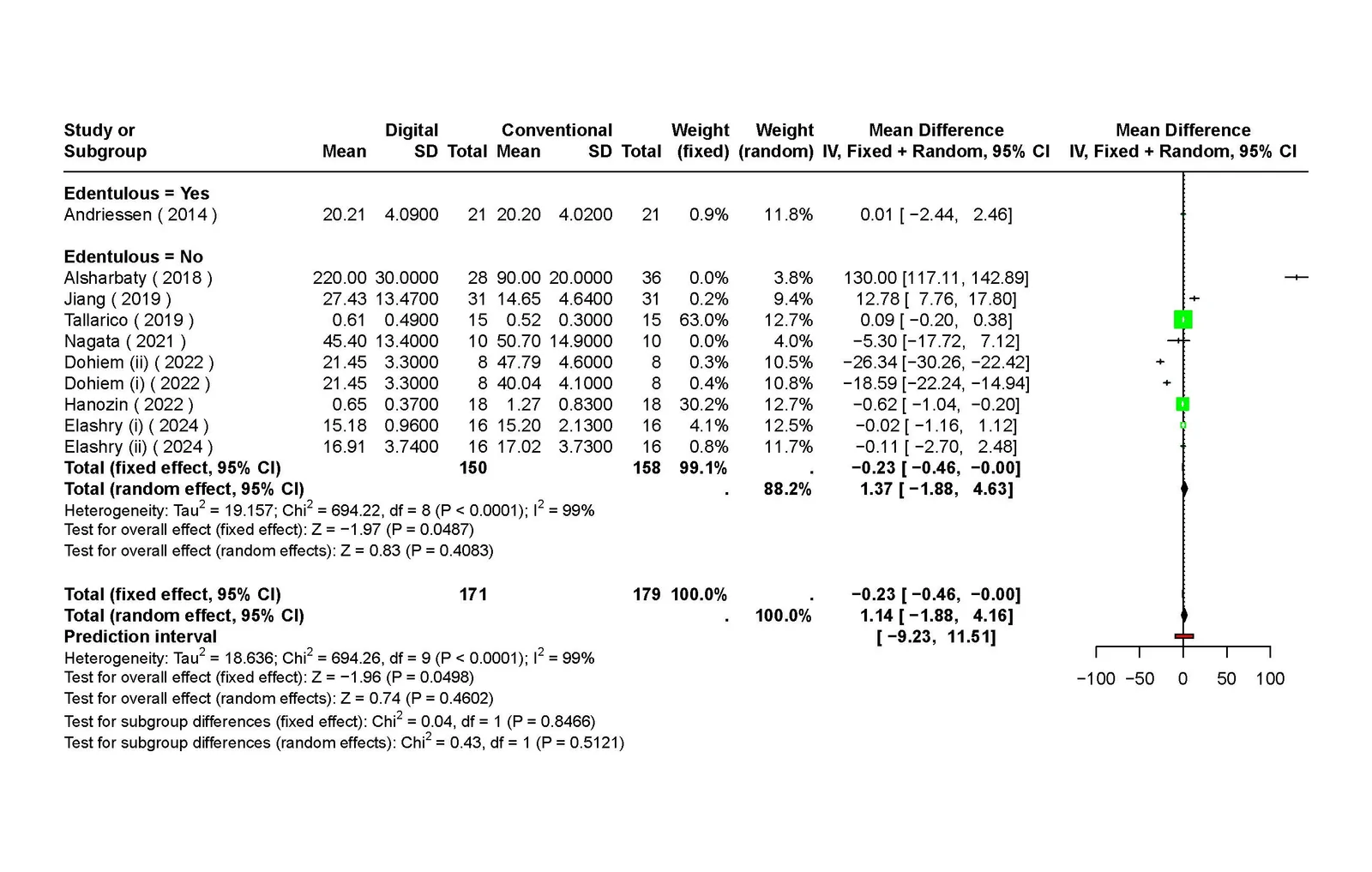
Figure 3. Forest plot of distance deviation based on full vs partial edentulism

**Figure-4:**
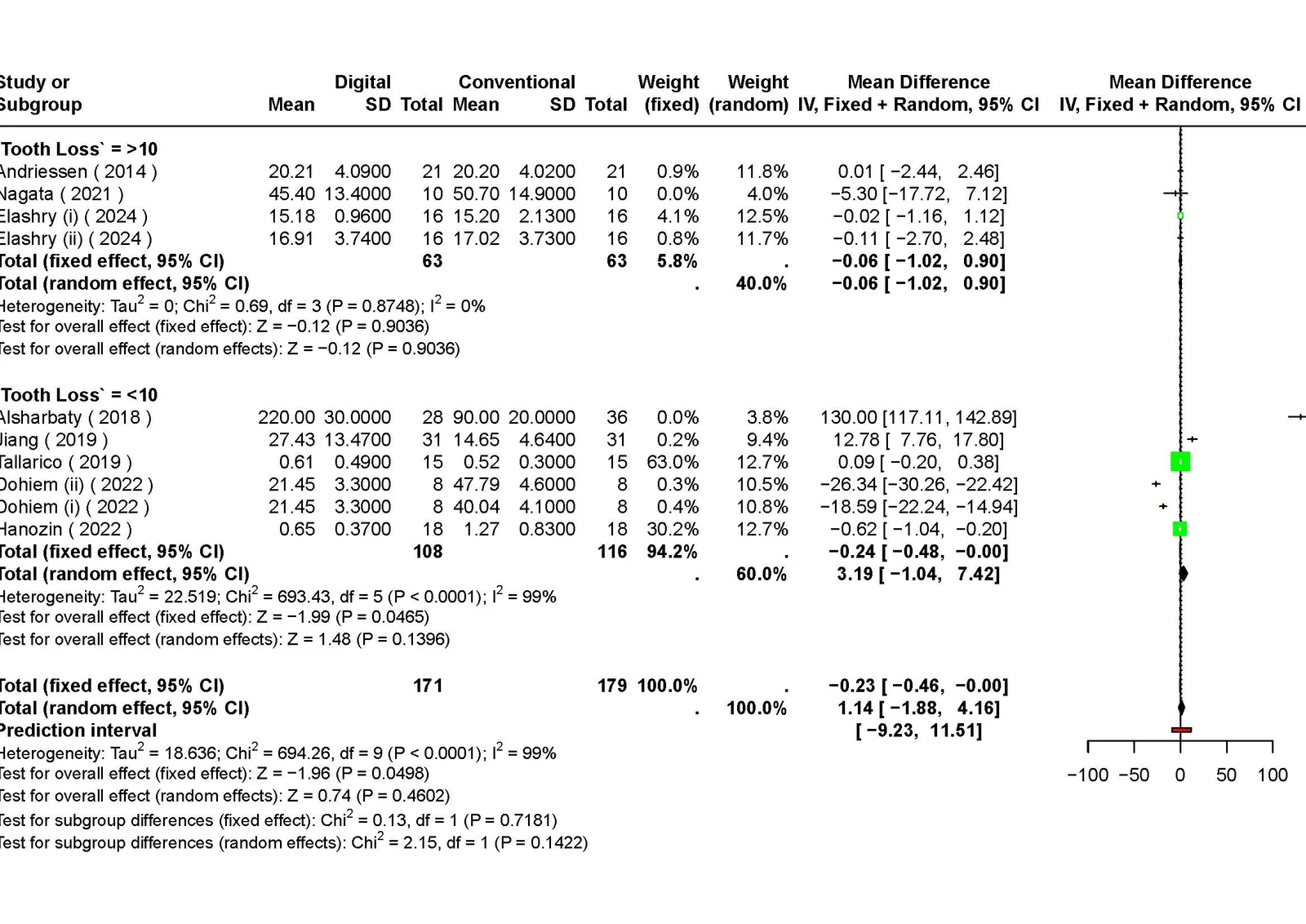
Figure 4. Forest plot of distance deviation based on number of lost teeth

**Figure-5:**
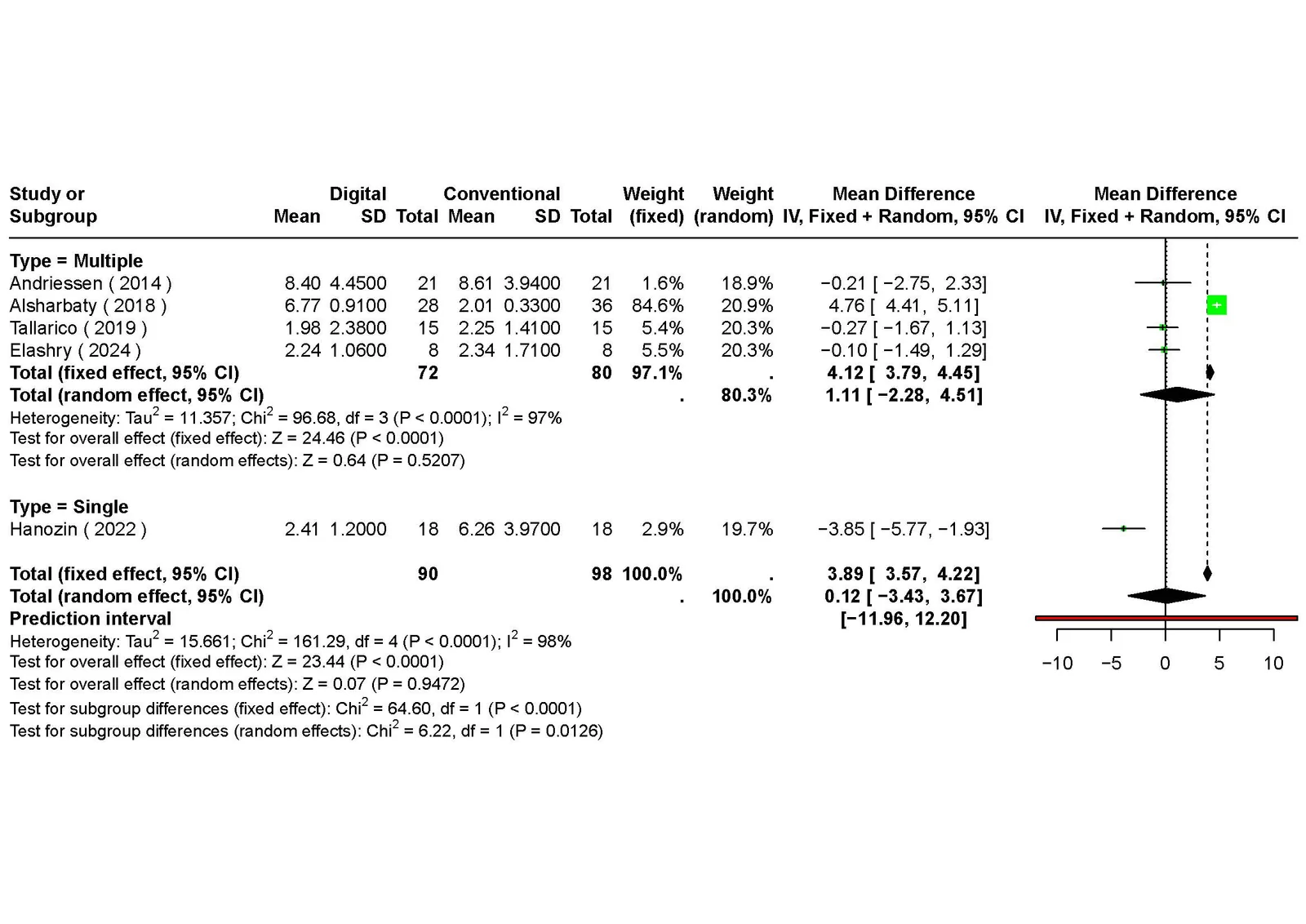
Figure 5. Forest plot of Angular deviation based on number of implants

**Figure-6:**
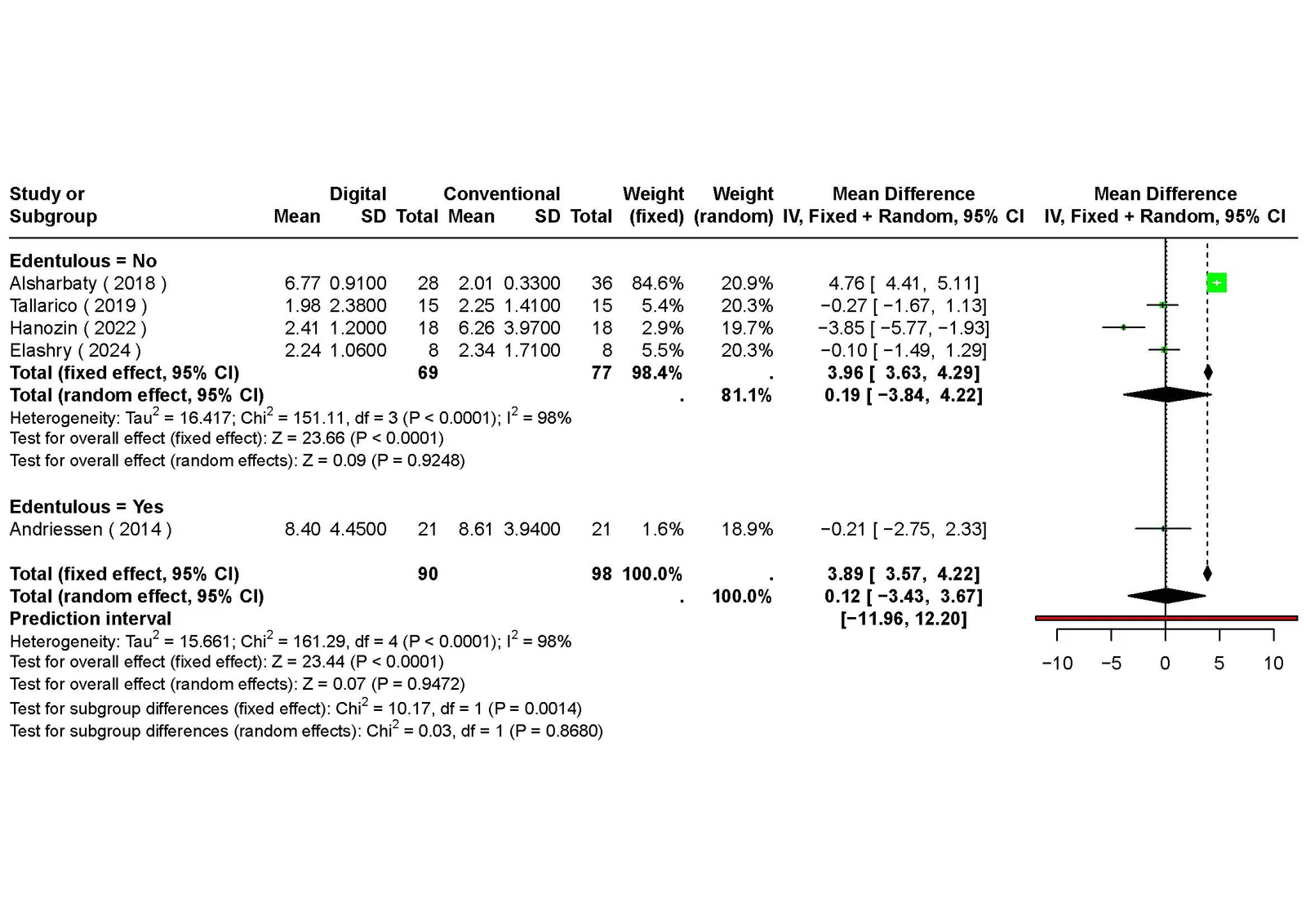
Figure 6. Forest plot of angular deviation based on full vs partial edentulism

**Figure-7:**
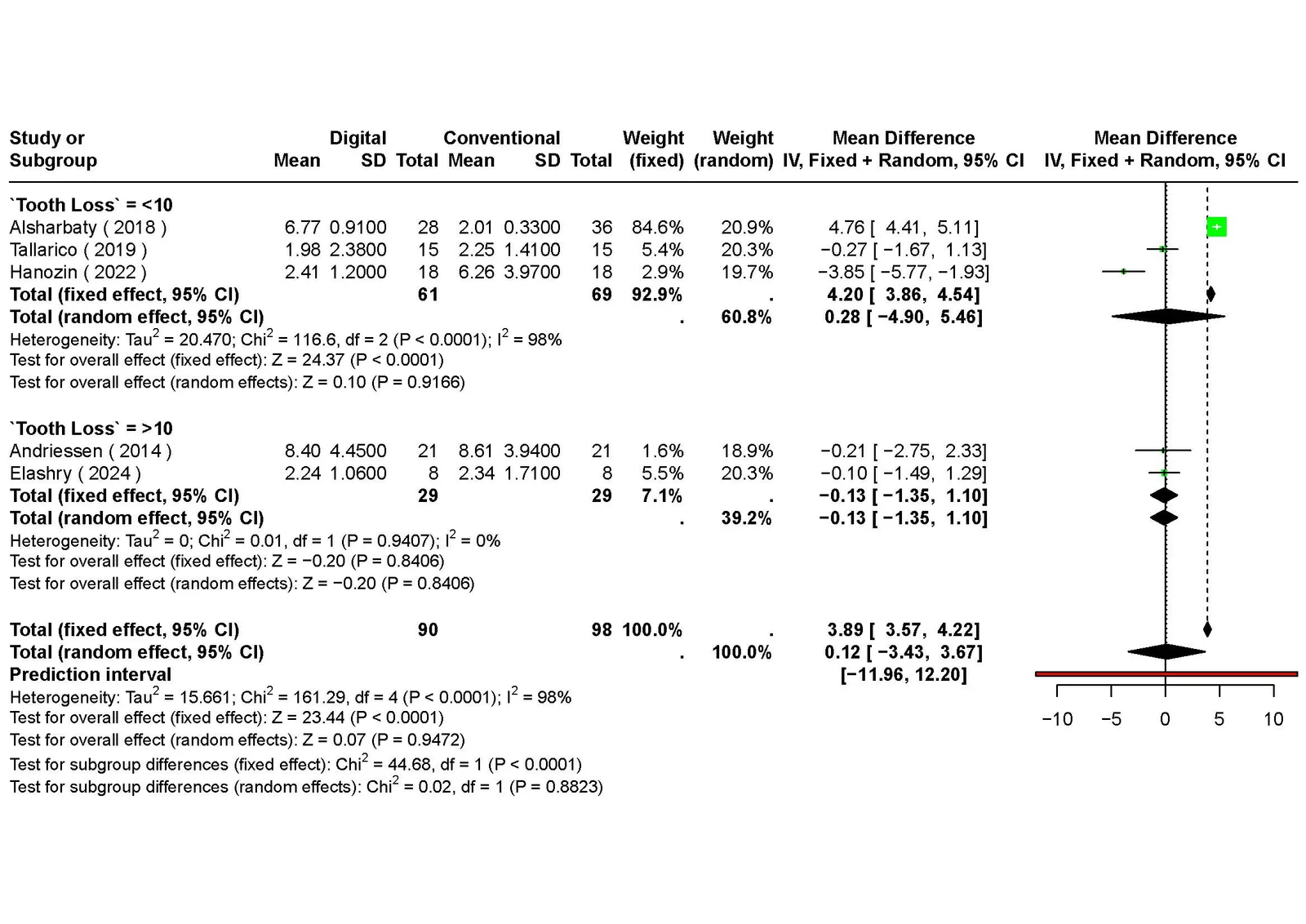
Figure 7. Forest plot of angular deviation based on number of lost teeth

**Figure-8:**
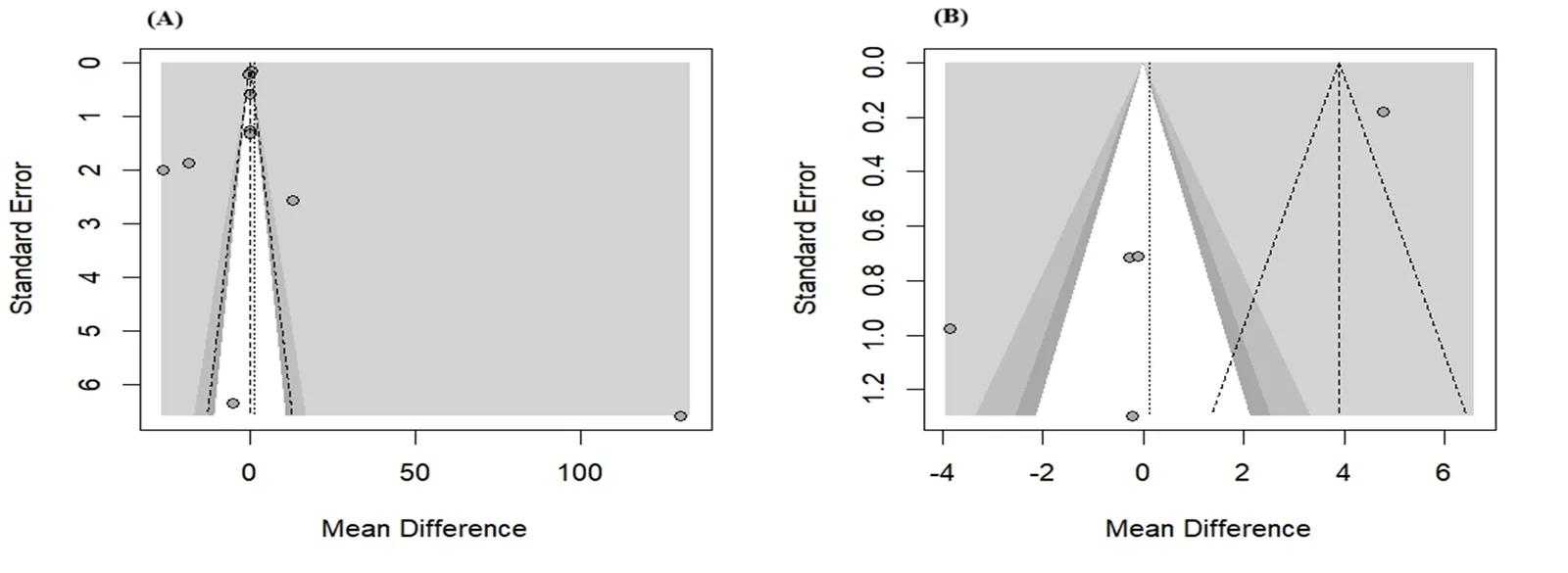
Figure 8. The funnel plots regarding publication bias assessment. Distance Deviation; (B) Angular Deviation

### Distance Deviation

A total of 8 clinical comparisons involving 171 digital and 179 conventional impressions were included in the quantitative synthesis for distance deviation. Under a fixed-effect model, digital impressions demonstrated a small but statistically significant reduction in distance deviation compared with conventional impressions (MD = −0.23 mm; 95% CI −0.46 to −0.00; P = 0.0498). However, between-study heterogeneity was extreme (τ² = 18.64; I² = 99%), indicating substantial inconsistency across studies. When a random-effects model was applied to account for heterogeneity, the difference between digital and conventional impressions was no longer statistically significant (MD = 1.14 mm; 95% CI −1.88 to 4.16; P = 0.46). The wide confidence and prediction intervals suggested considerable uncertainty in the magnitude and direction of the true effect across clinical settings.

### Angular Deviation

Five comparisons comprising 90 digital and 98 conventional impressions were included for angular deviation. Using a fixed-effect model, digital impressions were associated with a significantly greater angular deviation compared with conventional impressions (MD = 3.89°; 95% CI 3.57 to 4.22; P < 0.0001). Heterogeneity was again very high (τ² = 15.66; I² = 98%). Under a random-effects model, the pooled estimate indicated no statistically significant difference between impression techniques (MD = 0.12°; 95% CI −3.43 to 3.67; P = 0.95), with wide confidence and prediction intervals reflecting marked variability among studies. Subgroup Analyses All subgroup analyses were exploratory because several subgroups included few comparisons and heterogeneity often remained substantial. Therefore, subgroup estimates were interpreted as hypothesis-generating rather than definitive evidence of superiority for either impression technique. Subgroup analysis revealed a statistically significant interaction based on the number of implants (P = 0.0177, random-effects). In single-implant cases, digital impressions demonstrated a significantly lower distance deviation compared with conventional impressions (MD = −0.63 mm; 95% CI −1.04 to −0.21). In contrast, in multiple-implant cases, the random-effects model indicated a significantly greater distance deviation associated with digital impressions (MD = 6.95 mm; 95% CI 0.70 to 13.20), accompanied by substantial heterogeneity. A pronounced subgroup effect was also observed for angular deviation (P = 0.0126, random-effects). In multiple-implant rehabilitations, digital impressions were associated with greater angular deviation compared with conventional impressions (MD = 1.11°; 95% CI −2.28 to 4.51), although this difference did not reach statistical significance under random-effects assumptions. Conversely, in single-implant cases, digital impressions showed a significantly lower angular deviation (MD = −3.85°; 95% CI −5.77 to −1.93), based on a single included study. This single-implant angular-deviation finding was based on one study and should therefore be interpreted with particular caution.

### Publication Bias

Funnel plots for distance deviation and angular deviation are presented in Figure-8. Because fewer than 10 comparisons were available for each primary outcome, funnel-plot asymmetry could not be interpreted reliably and Egger’s test was not performed. Therefore, no firm conclusion can be made regarding publication bias or small-study effects. In patients with partial edentulism, the fixed-effect model showed a small but statistically significant reduction in distance deviation with digital impressions (MD = −0.23 mm; 95% CI −0.46 to −0.00), whereas the random-effects model did not confirm this finding (MD = 1.37 mm; 95% CI −1.88 to 4.63). In complete edentulism, no difference between impression techniques was observed (MD = 0.01 mm; 95% CI −2.44 to 2.46). No statistically significant subgroup interaction was detected (P = 0.51, random-effects). For angular deviation, fixed-effect pooling suggested a significantly greater angular deviation with digital impressions in partially edentulous patients (MD = 3.96°; 95% CI 3.63 to 4.29), while no significant difference was observed in completely edentulous patients (MD = −0.21°; 95% CI −2.75 to 2.33). However, under the random-effects model, no significant subgroup difference was identified (P = 0.87), reflecting substantial heterogeneity and limited data for complete edentulism. Among patients with fewer than 10 missing teeth, fixed-effect analysis showed a significant reduction in distance deviation with digital impressions (MD = −0.24 mm; 95% CI −0.48 to −0.00). This effect was not maintained under the random-effects model (MD = 3.19 mm; 95% CI −1.04 to 7.42). In patients with more than 10 missing teeth, no significant difference between impression techniques was observed. No statistically significant subgroup interaction was detected (P = 0.14, random-effects). For angular deviation, fixed-effect analysis demonstrated a significantly greater angular deviation associated with digital impressions in patients with fewer than 10 missing teeth (MD = 4.20°; 95% CI 3.86 to 4.54), whereas no difference was observed in patients with more than 10 missing teeth. These subgroup effects were not confirmed under the random-effects model (P = 0.88), again reflecting high heterogeneity.

## Discussion

This systematic review and meta-analysis aimed to compare the 3-D accuracy of digital and conventional implant impressions in partially and completely edentulous patients, using distance and angular deviation as primary outcomes and exploring three clinical modifiers (single vs multiple implants, partial vs complete edentulism, and <10 vs >10 missing teeth). Overall, when high between-study heterogeneity was appropriately accounted for with random-effects models, there was no statistically significant difference between digital and conventional impressions for either distance or angular deviation, and most subgroup effects based on edentulous status or extent of tooth loss were not robust. The only consistent pattern was observed in single-implant situations, where digital impressions tended to show smaller angular deviations (better accuracy) than conventional impressions, while in multiple-implant and more complex cases the direction and magnitude of the effect varied widely across studies, with some suggesting greater deviation for digital techniques. These findings suggest that, in clinical practice, digital impressions can achieve accuracy comparable to conventional methods overall and may offer a particular advantage in single-implant scenarios, but the evidence remains heterogeneous and context-dependent for extensive, multi-implant reconstructions.

Our findings of no overall statistically significant difference in distance or angular deviation between digital and conventional implant impressions, once heterogeneity is accounted for, are broadly in line with several recent reviews that describe comparable accuracy between the two modalities in clinical settings. Multiple systematic and umbrella reviews have concluded that intraoral scanners generally achieve accuracy within clinically acceptable limits and are often similar, or slightly superior, to conventional impressions in single-unit and short-span restorations, while results become more inconsistent for full-arch rehabilitations [18]. In vitro syntheses also frequently report a tendency for digital impressions to perform at least as well as conventional techniques in terms of trueness, precision, and angular deviation, although the magnitude of benefit varies by scanner and experimental setup [19]. At the same time, other reviews focusing on full-arch or highly complex implant cases emphasize conflicting results and substantial heterogeneity, with some evidence that conventional splinted open-tray impressions or photogrammetry may still be more reliable for extensive frameworks [20]. The pattern observed in our subgroup analyses—digital impressions performing better in single-implant conditions but not clearly superior in multiple-implant or large edentulous spans—mirrors the broader literature, which consistently describes digital workflows as predictable for simpler rehabilitations yet more technique- and case-sensitive for long-span and full-arch implant reconstructions [21].

The lack of a strong overall difference between digital and conventional impressions in our meta-analysis is plausible when the complexity of the clinical reality is considered. The studies included in our review differed markedly in implant number and distribution, arch (maxilla vs mandible), angulation, type of connection, and reference standards used to measure deviation. Previous methodological reviews highlight that factors such as scan body design, scanning strategy, arch length, and implant angulation can substantially influence digital scan accuracy, especially in multi-implant and full-arch situations [22]. In single-implant cases, the scanned area is small, stitching errors are minimal, and the main sources of error (impression material shrinkage, tray distortion, plaster expansion) are eliminated; this provides a rational explanation for the more favorable angular accuracy of digital impressions observed in our single-implant subgroup, a trend also reported in experimental and clinical work [23]. In contrast, with multiple implants and particularly longer spans, digital scans rely on repeated image stitching across a larger field, so any minor inaccuracies accumulate and may result in larger distance or angular deviations; simultaneously, conventional splinted techniques also have their own sources of distortion, leading to the highly heterogeneous and sometimes contradictory outcomes seen both in our analysis and in previous meta-analyses [24]. Additional variation in scanner generation and software, operator experience, and calibration protocols across the included studies likely further amplified heterogeneity, which explains why fixed-effect models sometimes suggested clear differences, whereas random-effects models—more appropriate for such diverse data—did not confirm a consistent advantage for either impression technique [25].

Even though our quantitative synthesis did not show a universal superiority of digital impressions in terms of linear or angular accuracy, the broader evidence demonstrates several important advantages of digital workflows that support their use in appropriate clinical contexts. Digital impressions are repeatedly associated with improved patient comfort, as they avoid impression trays and materials that can trigger gagging, nausea, or breathing discomfort, and patients often express a preference for intraoral scanning over conventional impressions [26, 27]. Digital workflows can reduce chairside time and streamline communication with the laboratory by enabling instant data transfer, eliminating shipping, and facilitating rapid design–manufacture cycles [28, 29]. The ability to immediately visualize the scan allows clinicians to detect voids, scan-body misalignment, or missing areas during the same appointment, potentially reducing remakes and adjustments. Moreover, digital data can be easily archived, duplicated, and integrated with CBCT or facial scans, enabling prosthetically driven implant planning and guided surgery [30, 31].

From a clinical standpoint, our findings support the use of digital impressions as a first-line option in single-implant and short-span reconstructions, where both our results and prior reviews indicate that intraoral scanners can achieve accuracy comparable or superior to conventional methods [32, 33, 34]. In these indications, the additional benefits in workflow efficiency, patient experience, and digital integration are likely to outweigh any small differences in 3-D deviation. For multiple implants and extensive edentulous spans, the lack of a consistent advantage and the wide prediction intervals in our meta-analysis suggest that clinicians should adopt a more individualized approach. In complex full-arch or highly angulated cases, current evidence still justifies the continued use of well-executed conventional splinted impressions, or the combination of digital scans with verification jigs or extraoral photogrammetry, until further robust clinical data confirm that full-arch intraoral scans can reliably match conventional accuracy [35, 36]. In everyday practice, this means selecting the impression technique not only based on technology availability but also on arch length, implant distribution, and the operator’s familiarity with digital protocols.

Clinical interpretation should distinguish statistical significance from clinical relevance. No universally accepted threshold defines an acceptable implant-impression error, and reported passive-fit limits vary across studies and clinical contexts. Commonly cited ranges for prosthetic misfit are approximately 60-150 micrometers, while some full-arch digital-impression studies have used stringent reference values around 100 micrometers and 0.4 degrees. In the present review, the wide random-effects confidence intervals and prediction intervals mean that statistically nonsignificant pooled results may still include clinically relevant differences, particularly in long-span or full-arch implant rehabilitations. Conversely, statistically significant fixed-effect estimates should not be overinterpreted when heterogeneity is extreme. Clinical decisions should therefore consider not only the pooled mean difference but also prosthesis span, implant number and angulation, verification procedures, operator experience, and the likelihood that the observed deviation would affect passive fit or require chairside/laboratory adjustment.

This meta-analysis has several limitations that should be considered when interpreting the findings. The number of available clinical studies directly comparing digital and conventional impressions for implants was limited, especially for completely edentulous full-arch cases and for some subgroups, which reduces the power of subgroup analyses and increases the risk that estimates are influenced by single studies. The included trials varied in design, sample size, implant systems, arch location, scan bodies, and measurement protocols, contributing to the very high heterogeneity observed and making it difficult to isolate the effect of the impression technique from other confounding factors. Different reference standards were used to quantify distance and angular deviation, and cut-off values for clinical acceptability were not uniform across studies. Technological progress over time means that older scanners and materials included in some studies may not reflect the performance of current systems, and operator learning curves were rarely reported or controlled. Finally, our review focused on accuracy outcomes and did not pool patient-centered measures such as comfort, preference, or time efficiency, so the conclusions primarily address 3-D deviation and may underestimate the broader clinical advantages or disadvantages of each impression approach. Additional limitations include the absence of prospective protocol registration, the lack of a systematic grey-literature search, and the limited ability to assess publication bias because fewer than 10 studies were available for each primary outcome. The risk-of-bias assessment also showed that methodological quality varied across the included studies, which may have influenced both pooled and subgroup estimates.

## Conclusion

Within the limitations of the available clinical evidence, digital and conventional implant impressions showed broadly comparable three-dimensional accuracy when random-effects models were used as the primary analysis. However, the pooled estimates must be interpreted cautiously because heterogeneity was extreme, the number of included studies was small, and several subgroup findings were based on sparse data. Digital impressions may be a reasonable option for single-implant and short-span restorations, especially when their workflow and patient-comfort advantages are valued. For multiple-implant, highly angulated, long-span, or full-arch cases, the current evidence does not support a universal preference for either technique; technique selection should be individualized and supported by appropriate verification procedures. Further well-designed, adequately powered clinical studies with standardized accuracy measures, transparent risk-of-bias reporting, and clinically meaningful thresholds are needed.

## Conflict of Interest

The authors declare no conflict of interest.

## AI Disclosure Statement

During the preparation of this manuscript, the authors used ChatGPT, OpenAI company for language editing, grammar improvement, and liboberry.com for reference management. After its use, the authors thoroughly reviewed, verified, and revised all AI-assisted content to ensure accuracy and originality. The authors take full responsibility for the integrity and final content of the published article.
